# Blood pressure in relation to general and central adiposity among 500 000 adult Chinese men and women

**DOI:** 10.1093/ije/dyv012

**Published:** 2015-03-05

**Authors:** Zhengming Chen, Margaret Smith, Huaidong Du, Yu Guo, Robert Clarke, Zheng Bian, Rory Collins, Junshi Chen, Yijian Qian, Xiaoping Wang, Xiaofang Chen, Xiaocao Tian, Xiaohuan Wang, Richard Peto, Liming Li, for the China Kadoorie Biobank Collaborative Group

**Affiliations:** ^1^Clinical Trial Service Unit & Epidemiological Studies Unit (CTSU), Nuffield Department of Population Health, University of Oxford, Oxford, UK,; ^2^Chinese Academy of Medical Sciences, Beijing, China,; ^3^China National Centre for Food Safety Risk Assessment, Beijing, China,; ^4^Tongxiang Centre for Disease Control and Prevention (CDC), Zhejiang, China,; ^5^Maiji CDC, Gansu, China,; ^6^Sichuan Provincial CDC, Chengdu, China,; ^7^Qingdao CDC, Qingdao, China,; ^8^Hainan Provincial CDC, Haikou, China and; ^9^Department of Epidemiology & Biostatistics, Peking University, Beijing, China

**Keywords:** Adiposity, blood pressure, cross-sectional studies, epidemiology, Chinese

## Abstract

**Background:** Greater adiposity is associated with higher blood pressure. Substantial uncertainty remains, however, about which measures of adiposity most strongly predict blood pressure and whether these associations differ materially between populations.

**Methods:** We examined cross-sectional data on 500 000 adults recruited from 10 diverse localities across China during 2004–08. Multiple linear regression was used to estimate the effects on systolic blood pressure (SBP) of general adiposity [e.g. body mass index (BMI), body fat percentage, height-adjusted weight] vs central adiposity [e.g. waist circumference (WC), hip circumference (HC), waist-hip ratio (WHR)], before and after adjustment for each other. The main analyses excluded those reported taking any antihypertensive medication, and were adjusted for age, region and education.

**Results:** The overall mean [standard deviation (SD)] BMI was 23.6 (3.3) kg/m^2^ and mean WC was 80.0 (9.5) cm. The differences in SBP (men/women, mmHg) per 1SD higher general adiposity (height-adjusted weight: 6.6/5.6; BMI: 5.5/4.9; body fat percentage: 5.5/5.0) were greater than for central adiposity (WC: 5.0/4.3; HC: 4.8/4.1; WHR: 3.7/3.2), with a 10 kg/m^2^ greater BMI being associated on average with 16 (men/women: 17/14) mmHg higher SBP. The associations of blood pressure with measures of general adiposity were not materially altered by adjusting for WC and HC, but those for central adiposity were significantly attenuated after adjusting for BMI (WC: 1.1/0.7; HC: 0.3/−0.2; WHR: 0.6/0.6).

**Conclusion:** In adult Chinese, blood pressure is more strongly associated with general adiposity than with central adiposity, and the associations with BMI were about 50% stronger than those observed in Western populations.

**Key Messages**

- Among the relatively lean Chinese adults who were not taking any blood pressure-lowering drugs, greater adiposity, irrespective how it was measured, was associated with higher blood pressure.
- Measures of general adiposity (e.g. BMI, body fat percentage) provided better prediction of blood pressure than measures of central adiposity (e.g. WC, HC, WHR).
- In this Chinese population, a 10 kg/m^2^ greater BMI was associated on average with 16 mmHg higher SBP, which was ∼50% stronger than observed in Western populations.
- Among a subset of individuals who were treated with antihypertensive therapies, the association of SBP with BMI was only ∼1/3 as strong as among those without any such treatments.

## Introduction

Controlled trials of weight loss interventions^1^^,^^2^ and Mendelian randomization studies of blood pressure in relation to adiposity-related genetic variants^3^^,^^4^ have both established the causal association between adiposity and blood pressure. Typically in Western adult populations, a 10 kg/m^2^ higher body mass index (BMI) is associated with ∼10 mmHg higher systolic blood pressure (SBP), throughout the range typically found in Western populations.^5^ However, there is little reliable evidence about whether these associations differ materially by age, sex or other personal characteristics. Likewise, there is still limited evidence about the strength of these associations in China, overall or in different population subgroups.^6–9^ These questions are particularly relevant to China, where high blood pressure is a leading cause of morbidity and premature mortality, mainly through its effects on stroke,^10–13^ and where levels of adiposity are increasingrapidly.^14^

Furthermore, there is conflicting evidence—both in China^6–9^^,^^15–19^ and elsewhere^18^^,^^20–27^—about which measures of adiposity are most strongly associated with blood pressure and whether these effects are modified substantially by age, gender and ethnicity.^28^ Some studies have reported that waist circumference (WC) and waist-hip ratio (WHR), both considered to be measures of central adiposity, are more strongly associated with blood pressure than BMI (considered a measure of general adiposity),^6^^,^^16^^,^^18^^,^^20^^,^^21^^,^^23^^,^^26^ but others have reported no material differences between them,^8^^,^^19^^,^^28^ inconsistent findings between men and women,^9^^,^^22^^,^^24^ or stronger association for BMI than for measures of central adiposity.^7^^,^^17^^,^^25^^,^^27^ Most previous studies have included too few participants or were limited by analysing blood pressure in dichotomized form only (i.e. those with or without hypertension) with little information about use of blood pressure-lowering treatment.^16^^,^^18^^,^^20^ Information on which, if any, measures of adiposity most strongly predict blood pressure may be clinically relevant, and might suggest whether general or central adiposity is a more fundamental determinant of blood pressure. For example, some suggested mechanisms by which adiposity could increase blood pressure probably depend chiefly on central adiposity (e.g. physical compression of the kidney,^29^^,^^30^ hyperinsulinaemia resulting from insulin resistance^29^^,^^31^ or systemic inflammation and oxidative stress leading to artery stiffness^31^^,^^32^), whereas other proposed mechanisms may depend chiefly on overall adiposity through dysfunction of adipose tissue (e.g. leptin and adiponectin secretion or activation of the rennin-angiotension-aldosterone system or sympathetic nervous system^29–31^).

We examine the associations of blood pressure with several different measures of adiposity among 500 000 adults in the China Kadoorie Biobank (CKB) study.^33^^,^^34^ In particular, we compared the strength of the associations of blood pressure with different measures of general and central adiposity, both independently and jointly, and whether these associations differed importantly in relevant population subgroups (e.g. by age, gender and prior medication).

## Methods

### Study population

Details of the CKB design, procedures and ethics approval have been reported elsewhere.^33^^,^^34^ Briefly, 512 891 participants (210 222 men, 302 669 women) aged 30–79 were recruited into the study from 10 localities (5 urban and 5 rural) in China during 2004–08. Potential eligible participants were identified through official residential records for the 100–150 administrative units (either rural villages or urban residential committees) within each of the 10 study areas. Invitation letters and study information leaflets were delivered to the eligible individuals by local community leaders or healthworkers after extensive publicity campaigns, and about 1 in 3 (33% in rural areas, 27% in urban areas) responded. All participants provided written informed consent and underwent a 60–75-min assessment at the local study clinics that included physical measurements, collection of blood sample and a computerized questionnaire administered by trained interviewers. The study procedures were standardized across the 10 regions, with regular calibration of measurement devices to ensure consistency of measurements.

### Adiposity measures

In this report six main adiposity variables, either directly measured or derived, were assessed, including height-adjusted weight, BMI, WC, hip circumference (HC), WHR and body fat percentage. All measurements were made once by trained technicians while participants were wearing light clothes (appropriate for the season) and no shoes. Standing height was measured to the nearest 0.1 cm using a stadiometer. Weight was measured to the nearest 0.1 kg using a body composition analyser (TANITA-TBF-300GS; Tanita Corporation), with subtraction of weight of clothing by 0.5 kg in summer, 1.0 kg in spring/autumn and 2.0–2.5 kg in winter. BMI was calculated as the weight in kilograms divided by the square of the height in metres (kg/m^2^). WC and HC were measured to the nearest 0.1 cm using a soft nonstretchable tape. WC was measured midway between the lowest rib and the iliac crest or, when this was not practicable, 1 cm above the umbilicus (in both cases, usually against bare skin, but subtracting 1 cm if on top of undergarments). HC was measured at the maximum circumference around the buttocks (usually over underpants, but subtracting 1 cm if over a skirt, or 2.5 cm if over trousers). WHR was the ratio of WC to HC. Body fat percentage was the fraction of total weight that was estimated to be fat weight by the Tanita body composition analyser using proprietary algorithms. In addition, other derived measures of adiposity were also considered separately, including fat mass (the product of body fat percentage and body weight), fat mass index (fat mass in kilograms divided by the square of height in metres, kg/m^2^), and waist-height ratio (WC divided by height).

### Blood pressure

Blood pressure was measured twice on the unclothed right upper arm using an automated A&D UA-779 digital monitor, recommended by the British Hypertension Society (www.bhsoc.org/bp-monitors), after participants had rested in the seated position for at least 5 min. Standard cuff size was used and if the difference between the two measurements was >10 mmHg, then a third measurement was taken withthe last two readings recorded. The mean values of the two recorded measurements were used for the analyses. The chief analyses reported are for SBP, as SBP is a better predictor of vascular mortality than diastolic blood pressure (DBP).^35^ Additional analyses for DBP for each measure of adiposity are reported in the supplementary material (available as Supplementary data at *IJE* online).

### Statistical methods

To limit effects of any possible measurement error, participants with extreme values of any adiposity measures (e.g. BMI < 15 kg/m^2^ or ≥40 kg/m^2^; *n* = 985) and blood pressures (e.g. SBP < 80 mmHg or ≥250 mmHg; *n* = 272) or missing data on body fat percentage (*n* = 241) were excluded. In addition, individuals who reported current use of ACE-inhibitors (*n* = 7133), beta-blockers (*n* = 6512), diuretics (*n* = 1452) or calcium antagonists (*n* = 14 132) were also excluded, except as shown in Figure 7. After all these exclusions, 486 936 (95%) participants remained for the main analyses.

Sex-specific correlations between different adiposity variables were calculated using Pearson partial correlation coefficients (r), adjusted for baseline age (8 categories) and area (10 categories).

**Figure 1.**
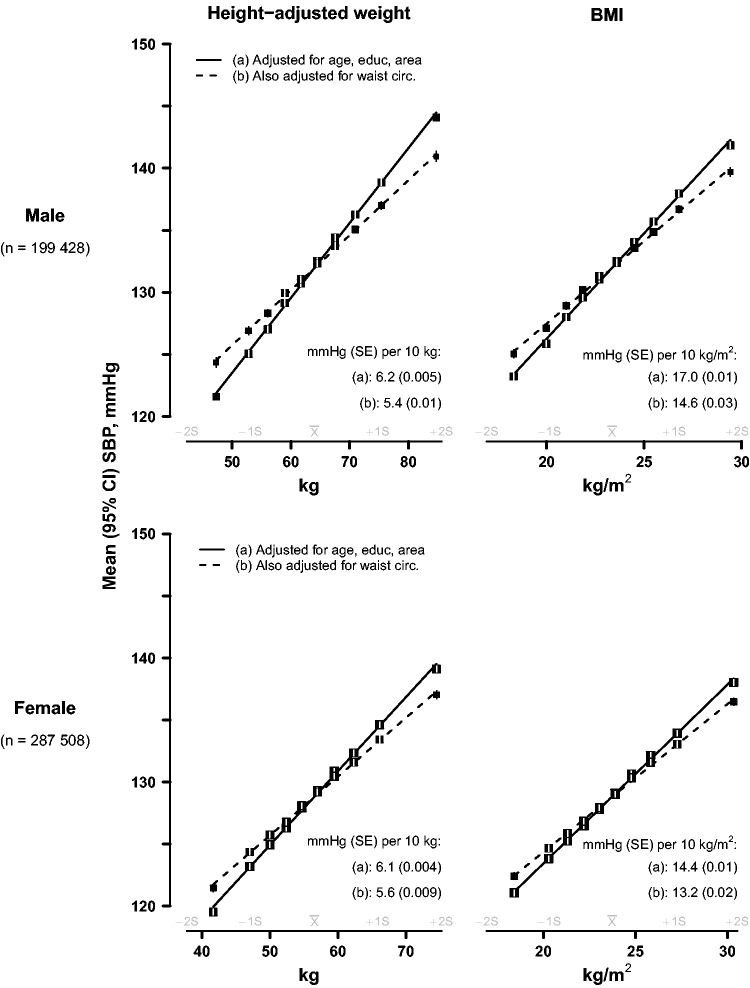
SBP vs height-adjusted weight and BMI, before and after adjustment for waist circumference, in men and women. The means of SBP were calculated for each sex-specific decile of height-adjusted weight (left panel) and BMI (right panel), with (a) standard adjustment for age, education and study area; and (b) additional adjustment for waist circumference (as a continuous variable). Closed squares represent the means of SBP with area inversely proportional to the variance of the mean SBP, and horizontal lines represent the corresponding 95% CIs.

**Figure 2.**
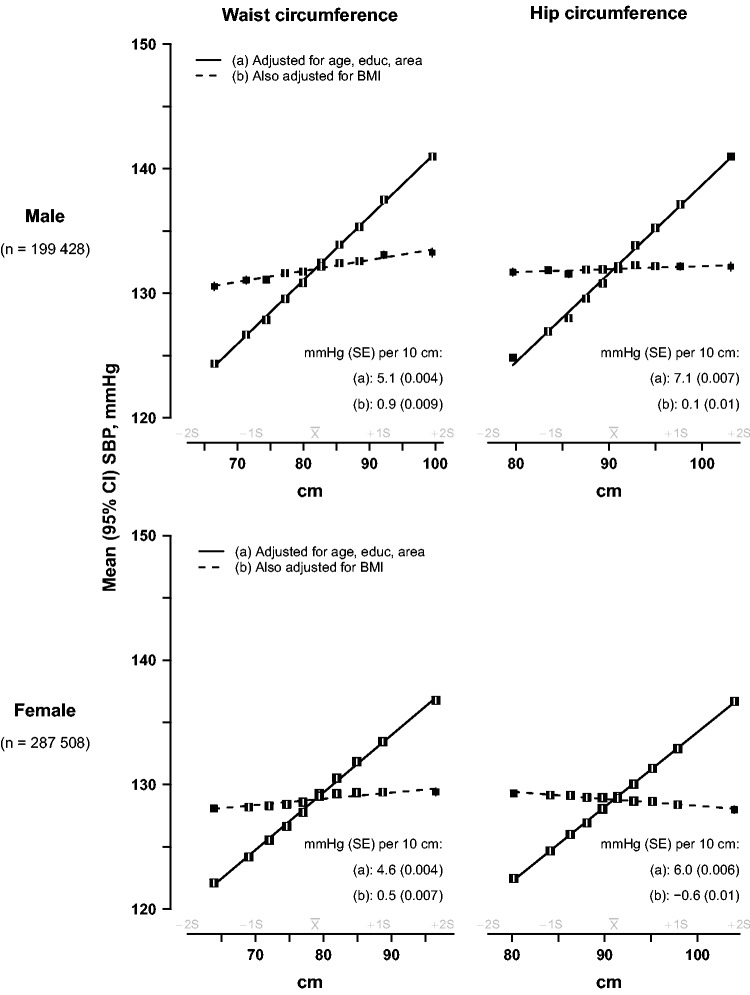
SBP vs waist and hip circumference, before and after adjustment for BMI, in men and women. The means of SBP were calculated for each sex-specific decile of waist circumference (left panel) and hip circumference (right panel), with (a) standard adjustment for age, education and study area; and (b) additional adjustment for BMI (as a continuous variable). Conventions as in Figure 1.

**Figure 3.**
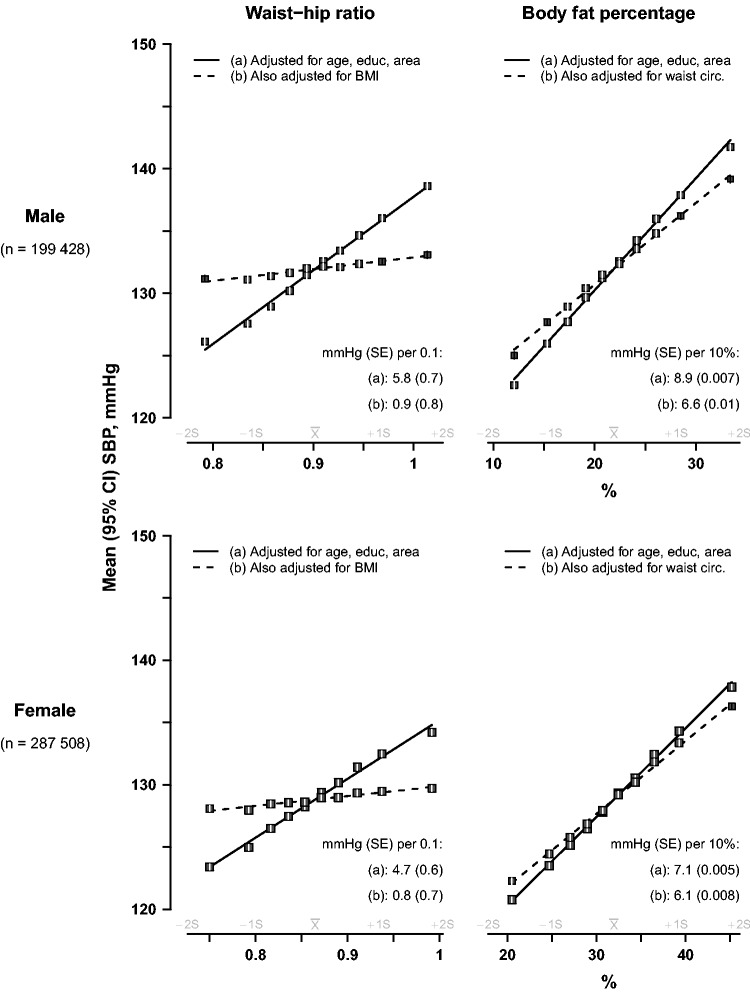
SBP vs waist-hip ratio and body fat percentage, before and after adjustment for BMI or waist circumference, in men and women. The means of SBP were calculated for each sex-specific decile of waist-hip ratio (left panel) and body fat percentage (right panel), with (a) standard adjustment for age, education and study area; and (b) additional adjustment for BMI for waist-hip ratio or waist circumference for body fat percentage. Conventions as in Figure 1.

For ‘categorical’ analyses (Figures 1–3, 7a), adjusted means of baseline SBP were calculated for each baseline sex-specific decile of each adiposity measure using multiple linear regression, with adjustment variously for baseline age (8 categories), education (6 categories), area (10 categories), WC (as a continuous variable) or BMI (continuous). For height-adjusted weight, standing height was also included in the model, in addition to weight categorized in deciles, as a continuous variable. Adjusted mean SBP for each decile was then plotted against the mean levels of the adiposity measure for that decile, together with the 95% confidence interval (CI). As all the resulting categorical associations were linear, a straight line was plotted through the categorical estimates using an inverse variance-weighted least squares fit method, and the slopes were reported as insets to each graph. To ensure that the slopes of these graphs are visually informative when comparing different adiposity measures, each horizontal axis is the same physical width and extends from −2 SD below to +2 SD above the mean level of the adiposity measure. As both the means and the SDs are sex-specific, and are never exactly the same in both sexes, the horizontal axes for men and womencover different ranges.

**Figure 4.**
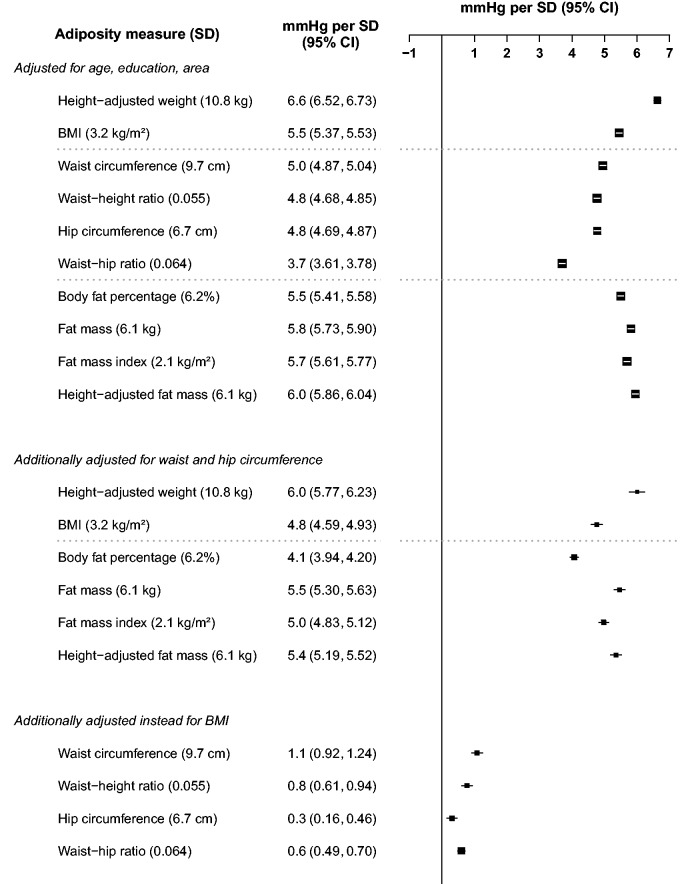
Higher SBP per standard deviation of each adiposity measure among men. The differences in SBP per 1 SD of each adiposity measure were calculated, with SBP regressed on level of each adiposity measure as a continuous variable. Adjustment for covariates is as above. Closed squares represent the mean differences in SBP with area inversely proportional to the variance of the SBP. Horizontal lines represent the corresponding 95% CIs.

**Figure 5.**
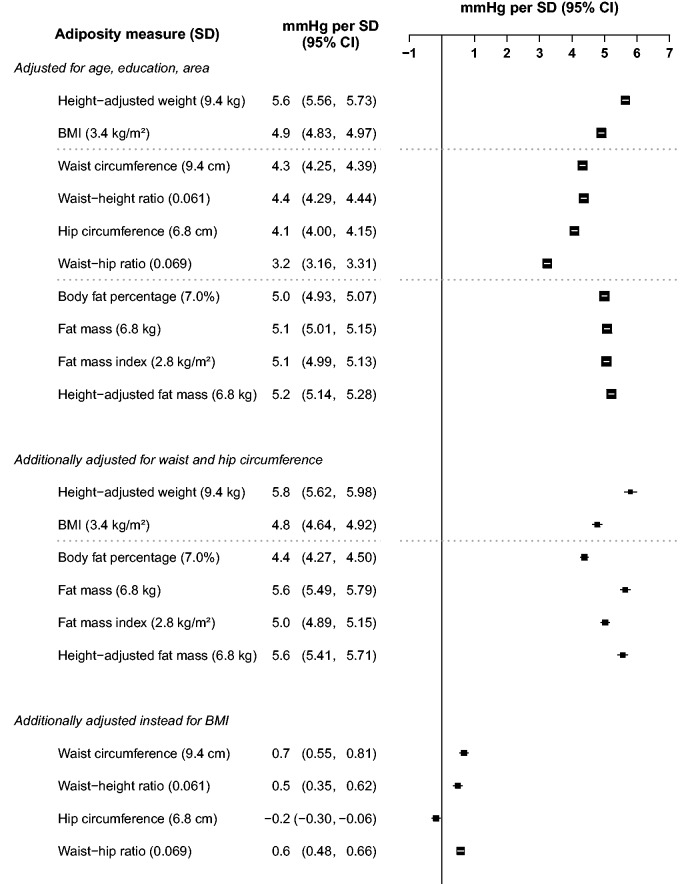
Higher SBP per standard deviation of each adiposity measure among women. Conventions as in Figure 4.

**Figure 6.**
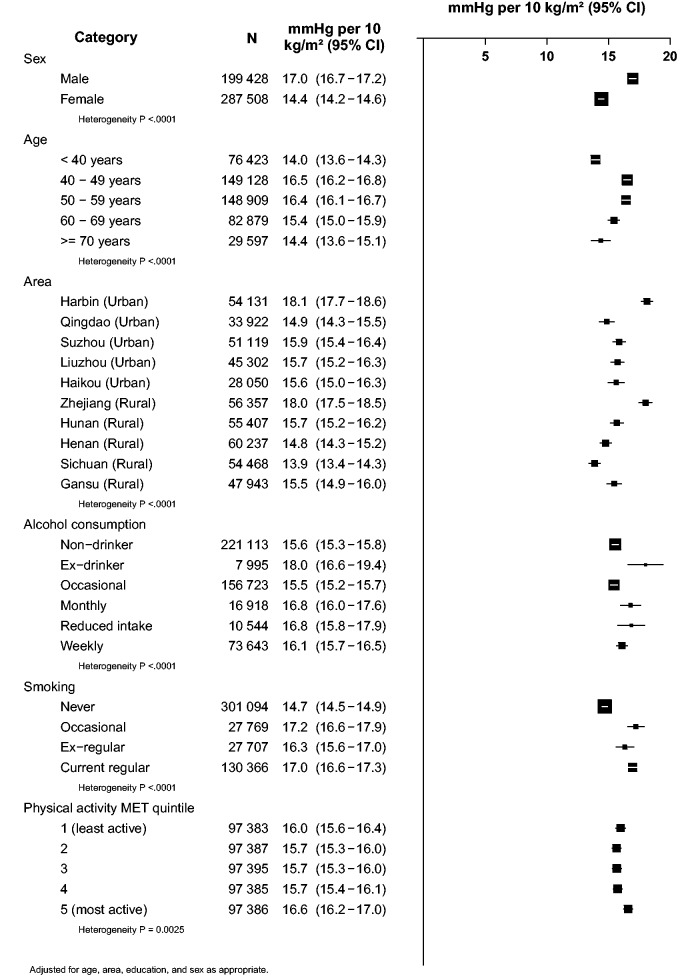
Higher SBP per 10 kg/m^2^ of BMI by different personal characteristics. Conventions as in Figure 3.

**Figure 7.**
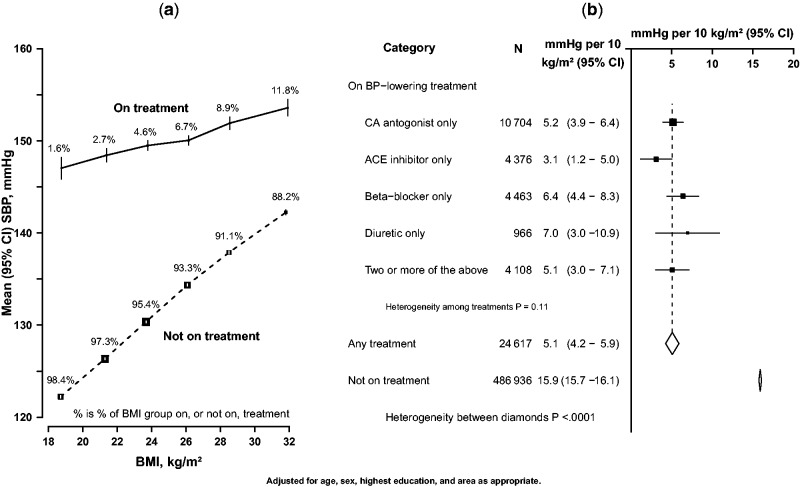
Association of SBP with BMI by treatment with blood pressure-lowering medication. (a) SBP vs BMI among individuals with or without treatment (conventions as in Figure 1); (b) differences in SBP per 10 kg/m^2^ of BMI by blood pressure-lowering treatment. Closed squares represent the mean differences in SBP and the horizontal lines represent the corresponding 95% CIs. The dotted vertical line indicates the overall mean difference in SBP among those using medication, and open diamonds indicates this and its 95% CI.

For continuous analyses (Figures 4–6, 7b), SBP was regressed on levels of each adiposity measure as a continuous variable using multiple linear regression, with adjustment for covariates as above and also, when appropriate, for HC (20 categories). Associations with ‘height-adjusted weight’ and with ‘height-adjusted fat mass’ were estimated by specifying body mass (weight) or fat mass as the (continuous) exposure variable, and adjusting for height (20 quantiles). Effect modification of continuous associations with BMI (Figure 6) was assessed by performing separate regressions within each level of the possible effect modifier. All analyses were carried out using SAS version 9.2, and R version 2.10.1 was used to graph results.

## Results

Among the included participants, the overall mean (SD) age was 51 (11) years, and the mean BMI was 23.6 (3.3) kg/m^2^, with only 4% having a BMI ≥ 30 kg/m^2^. The mean BMI was about 1 kg/m^2^ higher for individuals living in urban than in rural areas (24.2 vs 23.1 kg/m^2^), and slightly higher among women than men (23.7 vs 23.4 kg/m^2^). Among men, mean BMI was highest at 40–49 years, whereas among women it was highest at 50–59 years (Table 1). Body fat percentage showed similar patterns with age, and the mean levels were also greater in women (32.0%) than in men (21.9%) and likewise the absolute fat mass was also greater in women than in men (7.8 vs 5.3 kg), despite women weighing on average about 7.5 kg less.

**Table 1.** Distribution of anthropometric and blood pressure measures, by age and sex

		Mean (SD)^a^																	
Age, years	Participants	Height (cm)		Weight (kg)		BMI (kg/m^2^)		WC (cm)		HC (cm)		WHR		Body fat %age		SBP (mmHg)		DBP (mmHg)	
Male																			
30–39	29044	168	(6.2)	66.6	(11.0)	23.6	(3.3)	82.2	(9.4)	91.3	(6.7)	0.90	(0.061)	23.2	(6.3)	126	(14)	77	(10)
40–49	57778	167	(6.3)	66.1	(10.6)	23.7	(3.2)	82.7	(9.4)	91.3	(6.6)	0.90	(0.062)	23.1	(6.1)	128	(17)	79	(11)
50–59	60598	165	(6.3)	63.5	(10.4)	23.4	(3.1)	81.5	(9.5)	90.2	(6.5)	0.90	(0.063)	21.7	(5.9)	132	(20)	80	(11)
60–69	37385	163	(6.2)	61.0	(10.4)	22.9	(3.2)	80.9	(10.0)	89.5	(6.9)	0.90	(0.067)	20.3	(6.0)	138	(22)	79	(11)
70–79	14623	163	(6.3)	59.7	(10.6)	22.5	(3.3)	80.9	(10.5)	89.8	(7.4)	0.90	(0.069)	19.4	(6.2)	142	(22)	77	(11)
Total	199 428	165	(6.5)	64.0	(10.8)	23.4	(3.2)	81.8	(9.7)	90.5	(6.7)	0.90	(0.064)	21.9	(6.2)	132	(20)	79	(11)
Female																			
30–39	47379	156	(5.7)	55.9	(8.6)	23.0	(3.1)	75.3	(8.1)	90.0	(6.0)	0.84	(0.062)	30.7	(6.6)	118	(15)	73	(10)
40–49	91350	156	(5.8)	57.8	(8.9)	23.8	(3.2)	78.0	(8.6)	91.5	(6.3)	0.85	(0.064)	32.1	(6.6)	124	(18)	76	(11)
50–59	88311	154	(5.7)	56.9	(9.4)	24.0	(3.4)	80.1	(9.4)	91.2	(6.9)	0.88	(0.068)	32.6	(7.1)	132	(21)	78	(11)
60–69	45494	152	(5.7)	55.0	(9.9)	23.8	(3.7)	80.8	(10.3)	90.8	(7.6)	0.89	(0.072)	32.1	(7.7)	140	(23)	77	(11)
70–79	14974	150	(5.8)	52.7	(10.1)	23.4	(3.8)	80.6	(10.9)	90.4	(8.1)	0.89	(0.076)	31.2	(8.0)	144	(23)	75	(11)
Total	287 508	154	(6.0)	56.5	(9.4)	23.7	(3.4)	78.8	(9.4)	91.0	(6.8)	0.86	(0.069)	32.0	(7.0)	129	(21)	76	(11)

BMI, body mass index, WC, waist circumference, HC, hip-circumference, WHR, waist-hip ratio, body fat %age, body fat percentage; SBP, systolic blood pressure; DBP, diastolic blood pressure.

^a^Unadjusted means.

In contrast, mean (SD) WC was greater in men than in women [81.8 (9.7) cm vs 78.8 (9.4) cm], but it decreased somewhat with age in men and increased strongly with age in women, such that mean WC in old age was approximately similar between men and women (Table 1). Mean (SD) HC was 90.5 (6.7) cm in men and 91.0 (6.8) in women, and showed no consistent trend with age in either sex. Mean (SD) WHR was about 0.90 (0.06) at all age groups in men, but increased steadily from 0.84 (0.06) in the youngest to about 0.89 (0.08) in the oldest women.

In both sexes, mean SBP levels increased monotonically with age—by 16 mmHg over four decades from 30–39 years to 70–79 years in men, and by 26 mmHg in women. Mean DBP had an inverted U-shape association with age, with the levels increasing until age 55 years and declining thereafter. The overall mean SBP/DBP levels were about 3 mmHg higher in men (132/79 mmHg) than in women (129/76 mmHg).

BMI, WC and HC were all highly intercorrelated, with all correlation coefficients for pairwise comparisons in the range 0.76–0.86 (Table 2). Body fat percentage was correlated strongly with BMI (r = 0.80–0.89), less strongly with WC (0.74–0.78) and less strongly still with HC (0.62–0.70). WHR was strongly correlated with WC (0.80–0.82) but more modestly correlated with these other variables.

**Table 2.** Correlations coefficients^a^ between adiposity and blood pressure measures

		Weight	BMI	WC	HC	WHR	Body fat %age	SBP	DBP
Height	Male:	0.53	0.07	0.27	0.42	0.02	0.04	0.04	0.08
	Female:	0.48	0.03	0.20	0.37	−0.04	0.00	0.03	0.05
Weight		Male:	0.88	0.85	0.84	0.55	0.70	0.26	0.27
		Female:	0.89	0.83	0.85	0.47	0.78	0.24	0.25
BMI			Male:	0.86	0.76	0.63	0.80	0.28	0.28
			Female:	0.84	0.78	0.55	0.89	0.26	0.25
WC				Male:	0.81	0.82	0.74	0.24	0.25
				Female:	0.77	0.80	0.78	0.23	0.22
HC					Male:	0.33	0.62	0.21	0.21
					Female:	0.25	0.70	0.19	0.19
WHR						Male:	0.59	0.19	0.19
						Female:	0.54	0.17	0.16
Body fat %age							Male:	0.28	0.30
							Female:	0.27	0.27
SBP								Male:	0.74
								Female:	0.74

^a^Pearson partial correlation coefficients, adjusted for area and 5-year age group.

### Height-adjusted weight and body mass index

Figure 1 shows the sex-specific associations of SBP with height-adjusted weight, and BMI. After basic adjustment (solid lines), each extra 10 kg of body weight at each given height was associated with ∼6 mmHg higher SBP, and each 10 kg/m^2^ higher BMI was associated with 17 mmHg higher SBP in men and 14–15 mmHg higher SBP in women. After additional adjustment (dashed lines) for WC, the strengths of these associations were attenuated by 13–14% in men and by 8% in women.

### Waist and hip circumference

Figure 2 shows the sex-specific associations of SBP with WC and HC. With basic adjustment, each 10 cm wider circumference for either variable was typically associated with about 5–7 mmHg higher SBP. However, after further adjustment for BMI, all of these associations were largely or entirely eliminated. Upon taking account of BMI, the associations of blood pressure with WC were attenuated by 80–90% in both sexes, and for HC the remaining associations in women became slightly negative—i.e. greater HC was associated with slightly lower SBP.

### Waist-hip ratio and body fat percentage

Figure 3 shows the corresponding SBP associations for WHR and body fat percentage. With basic adjustment, a 0.1 cm higher WHR was associated with about 5–6 mmHg higher SBP, but with additional adjustment for BMI, the association was 80–85% shallower in both sexes. However, for body fat percentage, each extra 10 percentage points was associated with ∼9 mmHg higher SBP in males and ∼7 mmHg higher SBP in women after basic adjustment; and after additional adjustment for WC, the association with this measure of general adiposity was just 15% (female) or 25% (male) shallower.

### General and independent strengths of association

Figures 4 and 5 show the associations of SBP with different measures of adiposity expressed as the difference in SBP per 1 sex-specific SD higher level of adiposity. In both sexes, the single strongest predictor after basic adjustment was height-adjusted weight, with each 1 SD greater weight at any given height associated with 6.7 mmHg higher SBP in men, and 5.7 mmHg higher SBP in women. After additional adjustment for WC and HC, the positive association became slightly weaker in men and slightly stronger in women, such that in both sexes 1 SD greater weight-for-height was associated with about 6 mmHg higher SBP.

In both sexes, the next strongest associations after basic adjustment were for other measures of general adiposity: BMI, body fat percentage, fat mass, fat mass index and height-adjusted fat mass (all in the range of 5.5–6.0 mmHg per SD in men and 4.9–5.2 per SD in women), and the associations were only slightly attenuated by additional adjustment for WC and HC. After just basic adjustment, the associations for some measures of central or pelvic adiposity were nearly as strong as those for measures of general adiposity: WC, waist-height ratio and HC (4.8–5.0 mmHg per SD in men and 4.1–4.4 mmHg per SD in women). The association was substantially weaker for WHR (3.7 mmHg per SD in men, 3.2 mmHg per SD in women). However, all of these associations with WC, waist-height ratio, HC and WHR were again largely or wholly obliterated by additional adjustment for BMI.

### Effect modification for BMI

Height-adjusted weight was the strongest predictor of SBP, but this variable is not easy to apply in clinical practice. Of the next strongest predictors, BMI and body fat percentage may be clinically the most applicable, but BMI is the simpler of the two and does not require any technically advanced equipment. So, Figure 6 shows the strength of the association with BMI per 10 kg/m^2^ in more detail. There was only modest variation in the strength of the association across various population subgroups (about a fifth stronger in men than women and at age 40–59 than at age <40 years, and about a third stronger in some geographical areas than in others).

### Association in participants receiving blood pressure-lowering treatment

Figure 7 shows the association of SBP with BMI for both sexes combined among the 24 607 participants who were receiving some form of blood pressure-lowering treatment at baseline. Among them SBP was still linearly and positively associated with BMI, but the association was about two-thirds shallower: just 5 mmHg (Figure 7a, solid line) rather than 16 mmHg, overall higher SBP per 10 kg/m^2^ higher BMI (Figure 7a, dashed line). Figure 7b suggests that the strength of association may not have differed greatly by class of blood pressure-lowering medication, but there was only limited statistical power to detect any differences.

### Additional analyses

The associations of SBP with BMI and body fat percentage were somewhat, but not entirely, independent of each other, with adjustment of each association for the other factor generally attenuating the associations by 40–60% (Supplementary Figure 1, available as Supplementary data at *IJE* online). Mean adult SBP is substantially higher in China during winter than summer,^36^ but adjustment of the main SBP results for month of survey made no material difference (data not shown). Additional adjustment for pulse rate also did not alter the results. All of the main findings for SBP were also broadly applicable for DBP (Supplementary Figures 2–7, available as Supplementary data at *IJE* online).

## Discussion

In this cross-sectional study of nearly 500 000 Chinese adults, height-adjusted weight was the strongest predictor of blood pressure in both men and women, irrespective of whether the analyses were adjusted for other measures of adiposity. Among the widely used clinical measures of adiposity, BMI and body fat percentage were the next strongest predictors, and both were largely independent of each other. On average, a 10 kg/m^2^ greater BMI was associated with 16 mmHg higher SBP, much stronger than that observed previously in Western populations. WC and HC were each strongly positively associated with SBP, unless adjustment was made for BMI, when the association largely or entirely disappeared.WHR was a relatively poor predictor of SBP in this adult Chinese population.

Our study findings are consistent with those previously reported by several much smaller studies in China and elsewhere,^7^^,^^17^^,^^25^^,^^27^ but differ from those in several other studies^6^^,^^16^^,^^18^^,^^20^^,^^21^^,^^23^^,^^26^ which reported that measures of central adiposity (e.g. WC, WHR, waist-height ratio) were stronger predictors of blood pressure than BMI. The reasons for these inconsistencies are uncertain, but this study is much larger than any previous studies and uses measured blood pressure rather than history of hypertension. Moreover, the main analyses also excluded participants on blood pressure-lowering treatment.

The strength of associations of SBP with BMI were 50% stronger in this Chinese population compared with those previously reported in similarly large studies of mainly Western populations.^5^ These differences are too great to be accounted for by chance or by small differences in levels of adjustment for confounding. It is possible that relatively weak associations observed in the Western populations may be due chiefly to more widespread use of antihypertensive treatment, even though such information was not generally available in previous studies.^5^ In the present study, only about 14% of participants with hypertension were treated with any blood pressure-lowering therapies, and among them the association of SBP with BMI was about two-thirds shallower than among those without any such treatments. Since very few people were obese in our study population, it is unlikely that use of standard cuff size for measuring blood pressure would produce any biased association. However, it may also be possible that the strength of this relationship is not biologically universal but may differ geographically or with time because, for example, of interactions with other environmental (e.g. dietary patterns) or genetic determinants of adiposity, blood pressure or the relationship between adiposity and blood pressure.

The present study indicates that central adiposity is a less important determinant of blood pressure than is general adiposity, at least among Chinese adults—and, indeed, their associations with blood pressure may be largely or entirely because they are so highly correlated with measures of general adiposity. Weight-for-height was the best single predictor, but this variable is not easy to apply in clinical practice without a return to weight-for-height tables sometimes used in the past.^37^ BMI and body fat percentage were almost as good, and each added some independent predictive information to the other. These results suggest that adipose tissue in general may contribute to higher blood pressure, rather than just adipose tissue around the abdomen or, more particularly, in the intra-abdominal space. This may argue against the overall importance of proposed mechanisms that adiposity increases blood pressure mainly through physical compression of the kidney or systemic inflammation and oxidative stress, which depend importantly on the amount of intra-abdominal fat.^29^^,^^30^ Instead, other proposed mechanisms that are largely independent of intra-abdominal fat (such as dysfunction of adipose tissue, and activation of sympathetic nervous system) may play more important roles in causing high blood pressure.^29–31^ More accurate measures of intra-abdominal and subcutaneous fat (e.g. by imaging) in Chinese adults may further test this hypothesis. Treatment with any of the major classes of blood pressure-lowering medication appears to significantly attenuate these relationships. It can be inferred that, for any decrease in BMI, the associated lowering of blood pressure is less pronounced for those already receiving blood pressure-lowering medication.

Although general rather than central adiposity was clearly a better predictor of blood pressure in this adult Chinese population, there is well-established evidence, both in China^38–40^ and elsewhere,^41^^,^^42^ that measures of central adiposity (e.g. WC, WHR) are much stronger independent determinants of diabetes than is BMI. Blood pressure and diabetes (together with blood lipids) are major intermediate components of the causal pathways by which greater adiposity causes ischaemic heart disease (IHD)^5^ and stroke (perhaps especially ischaemic stroke),^12^^,^^14^^,^^43–45^ but their relative contribution to disease risks might differ between different populations, depending perhaps on the prevalence of diabetes and high blood pressure in the populations. Therefore, if BMI really is a particularly good predictor of blood pressure, and WC of diabetes, then the ability of BMI and WC to predict cardiovascular disease risk may also vary in different populations and over time, which may occur in Western populations as diabetes prevalence in middle age increases yet average blood pressure levels fall. The large prospective cohort studies that are now established in a wide range of populations should help to test these hypotheses.^34^^,^^46–48^ The independent contributions of general and central adiposity to different intermediate causal pathways may be why high BMI and high WC are each strongly associated with all-cause mortality in Western and Chinese populations,^5^^,^^44^^,^^45^^,^^47^^,^^49^^,^^50^ and why each adds independent information in the prediction of mortality^47^^,^^50^ despite being highly correlated with each other.

## Supplementary Data

Supplementary data are available at *IJE* online.

## Funding

Baseline survey: Kadoorie Charitable Foundation, Hong Kong. Long-term continuation: UK Wellcome Trust (088158/Z/09/Z, 104085/Z/14/Z), Chinese Ministry of Science and Technology (2011BAI09B01), Chinese National Natural Science Foundation (81390541). The British Heart Foundation, UK Medical Research Council and Cancer Research UK provide core funding to the Oxford CTSU.

## Supplementary Material

Supplementary Data

## China Kadoorie Biobank collaborative group:

International Steering Committee: Junshi Chen, Zhengming Chen, principal investigator (PI), Rory Collins, Liming Li (PI), Richard Peto. International Co-ordinating Centre, Oxford: Derrick Bennett, Yumei Chang, Yiping Chen, Zhengming Chen, Robert Clarke, Huaidong Du, Xuejuan Fan, Haiyan Gao, Simon Gilbert, Andri Iona, Rene Kerosi, Ling Kong, Om Kurmi, Garry Lancaster, Sarah Lewington, John McDonnell, Winnie Mei, Iona Millwood, Qunhua Nie, Jayakrishnan Radhakrishnan, Paul Ryder, Sam Sansome, Dan Schmidt, Paul Sherliker, Margaret Smith, Rajani Sohoni, Robin Walters, Jenny Wang, Lin Wang, Alex Williams, Ling Yang, Xiaoming Yang. National Co-ordinating Centre, Beijing: Zheng Bian, Ge Chen, Lei Guo, Yu Guo, Bingyang Han, Can Hou, Peng Liu, Jun Lv, Pei Pei, Shuzhen Qu, Yunlong Tan, Canqing Yu, Huiyan Zhou. Ten Regional Co-ordinating Centres: Qingdao Qingdao CDC: Zengchang Pang, Shaojie Wang, Yun Zhang, Kui Zhang. Licang CDC: Silu Liu, Wei Hou. Heilongjiang Provincial CDC: Zhonghou Zhao, Shumei Liu, Zhigang Pang. Nangang CDC: Weijia Feng, Shuling Wu, Liqiu Yang, Huili Han, Hui He, Bo Yu. Hainan Provincial CDC: Xianhai Pan, Shanqing Wang, Hongmei Wang. Meilan CDC: Xinhua Hao, Chunxing Chen, Shuxiong Lin, Xiangyang Zheng. Jiangsu Provincial CDC: Xiaoshu Hu, Minghao Zhou, Ming Wu, Ran Tao. Suzhou CDC: Yeyuan Wang, Yihe Hu, Liangcai Ma, Renxian Zhou, Guanqun Xu, Yan Lu. Guangxi Provincial CDC: Baiqing Dong, Naying Chen, Ying Huang. Liuzhou CDC: Mingqiang Li, Jinhuai Meng, Zhigao Gan, Jiujiu Xu, Yun Liu, Jingxin Qing. Sichuan Provincial CDC: Xianping Wu, Yali Gao, Ningmei Zhang. Pengzhou CDC: Guojin Luo, Xiangsan Que, Xiaofang Chen. Gansu Provincial CDC: Pengfei Ge, Jian He, Xiaolan Ren. Maiji CDC: Hui Zhang, Enke Mao, Guanzhong Li, Zhongxiao Li, Jun He, Yulong Lei, Xiaoping Wang. Henan Provincial CDC: Guohua Liu, Baoyu Zhu, Gang Zhou, Shixian Feng. Huixian CDC: Yulian Gao, Tianyou He, Li Jiang, Jianhua Qin, Huarong Sun. Zhejiang Provincial CDC: Liqun Liu, Min Yu, Yaping Chen, Ruying Hu. Tongxiang CDC: Zhixiang Hu, Jianjin Hu, Yijian Qian, Zhiying Wu, Chunmei Wang, Lingli Chen. Hunan Provincial CDC: Wen Liu, Guangchun Li, Huilin Liu. Liuyang CDC: Xiangquan Long, Xin Xu, Youping Xiong, Zhongwen Tan, Xuqiu Xie, Yunfang Peng, Weifang Jia.
