# Supplementary material for: Blood pressure in relation to general and central adiposity among 500 000 adult Chinese men and women
Source: Int J Epidemiol. 2015 Mar 5;44(4):1305–19. doi: 10.1093/ije/dyv012 (PMC4588860; doi:10.1093/ije/dyv012)
Supplement: Supplementary Data [file supp_44_4_1305__index.html]

Blood pressure in relation to general and central adiposity among 500 000 adult Chinese men and women — Blood pressure in relation to general and central adiposity among 500 000 adult Chinese men and women — Supplementary Data 

# Blood pressure in relation to general and central adiposity among 500 000 adult Chinese men and women

## Supplementary Data

files

**Files in this Data Supplement:**

- Supplementary Data - pdf file
