# Supplementary material for: Blood pressure in relation to general and central adiposity among 500 000 adult Chinese men and women
Source: Int J Epidemiol. 2015 Mar 5;44(4):1305–19. doi: 10.1093/ije/dyv012 (PMC4588860; doi:10.1093/ije/dyv012)
Supplement: Supplementary Data [file supp_dyv012_ije-2014-09-1084-File009.pdf]

Web figure 1. Systolic blood pressure versus BMI and body fat percentage, before (solid lines) and after (dashed lines) adjustment for each other

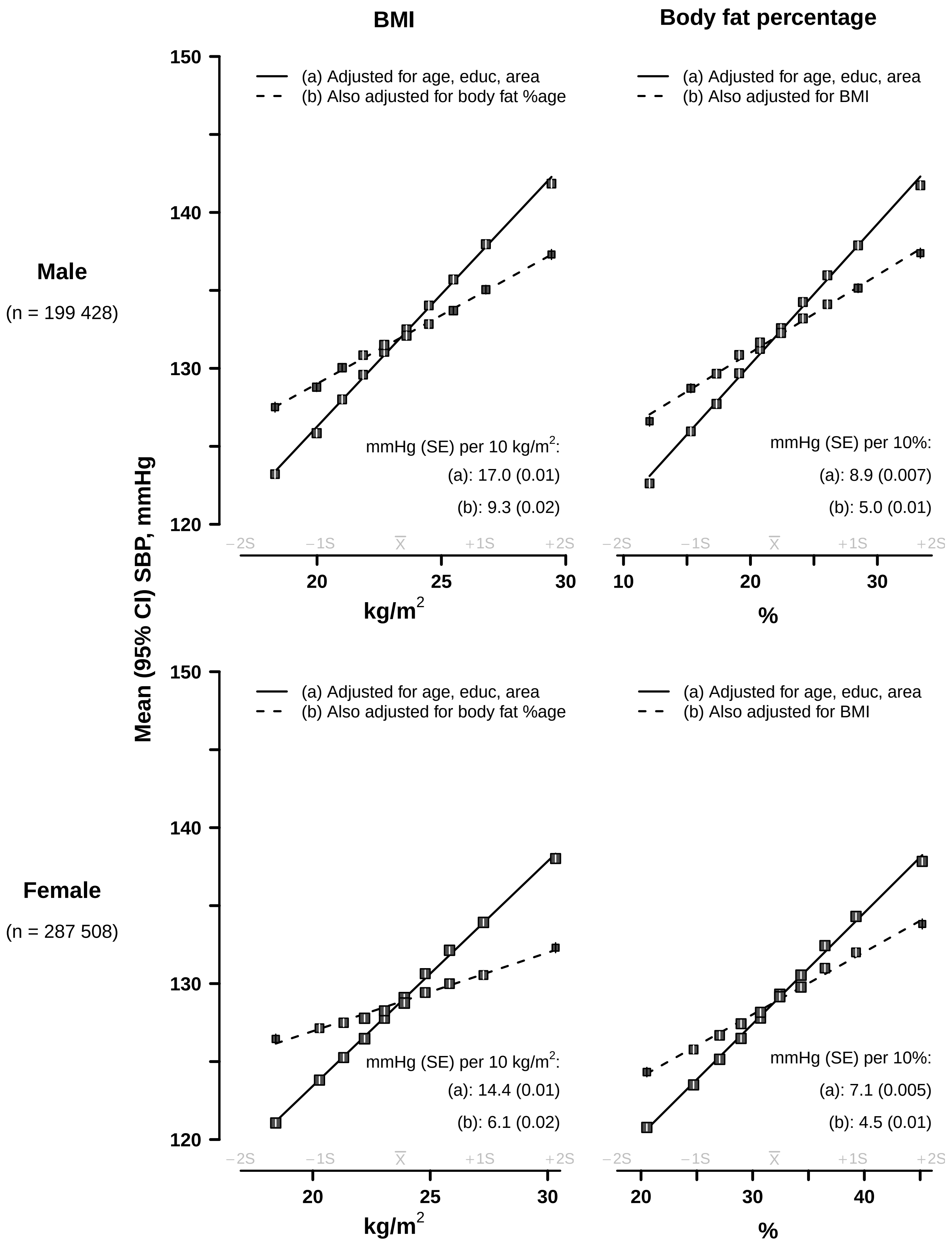

K:\kadoorie\Analysis\Adiposity Intermediate\Adip SBP\Outputs from meeting on\20140519\Web figure 1 SBP vs BMI Fatpct 19May2014.pdf

Web figure 2. Diastolic blood pressure versus height−adjusted weight and BMI, before (solid lines) and after (dashed lines) adjustment for waist circumference

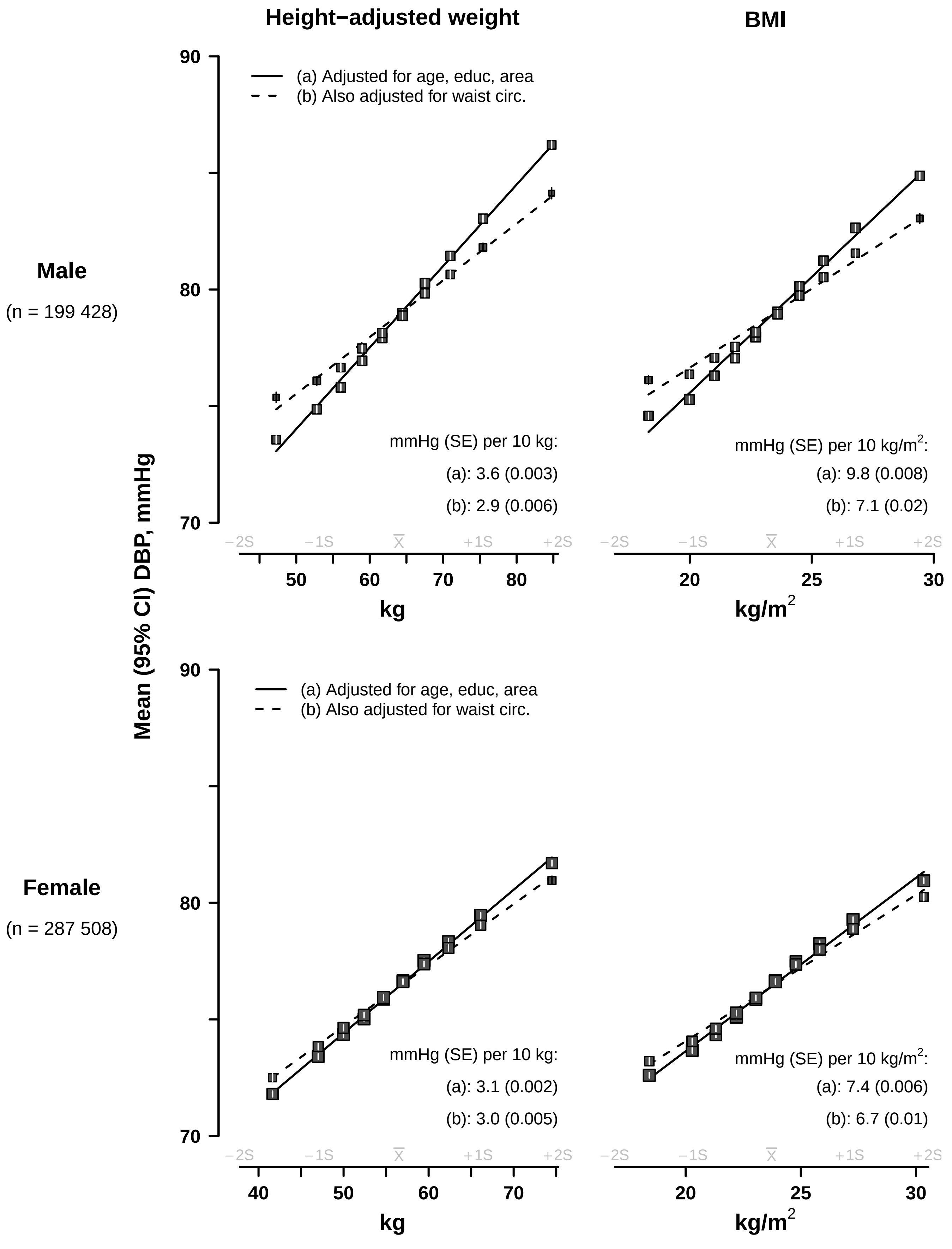

Web figure 3. Diastolic blood pressure versus waist and hip circumference, before (solid lines) and after (dashed lines) adjustment for BMI

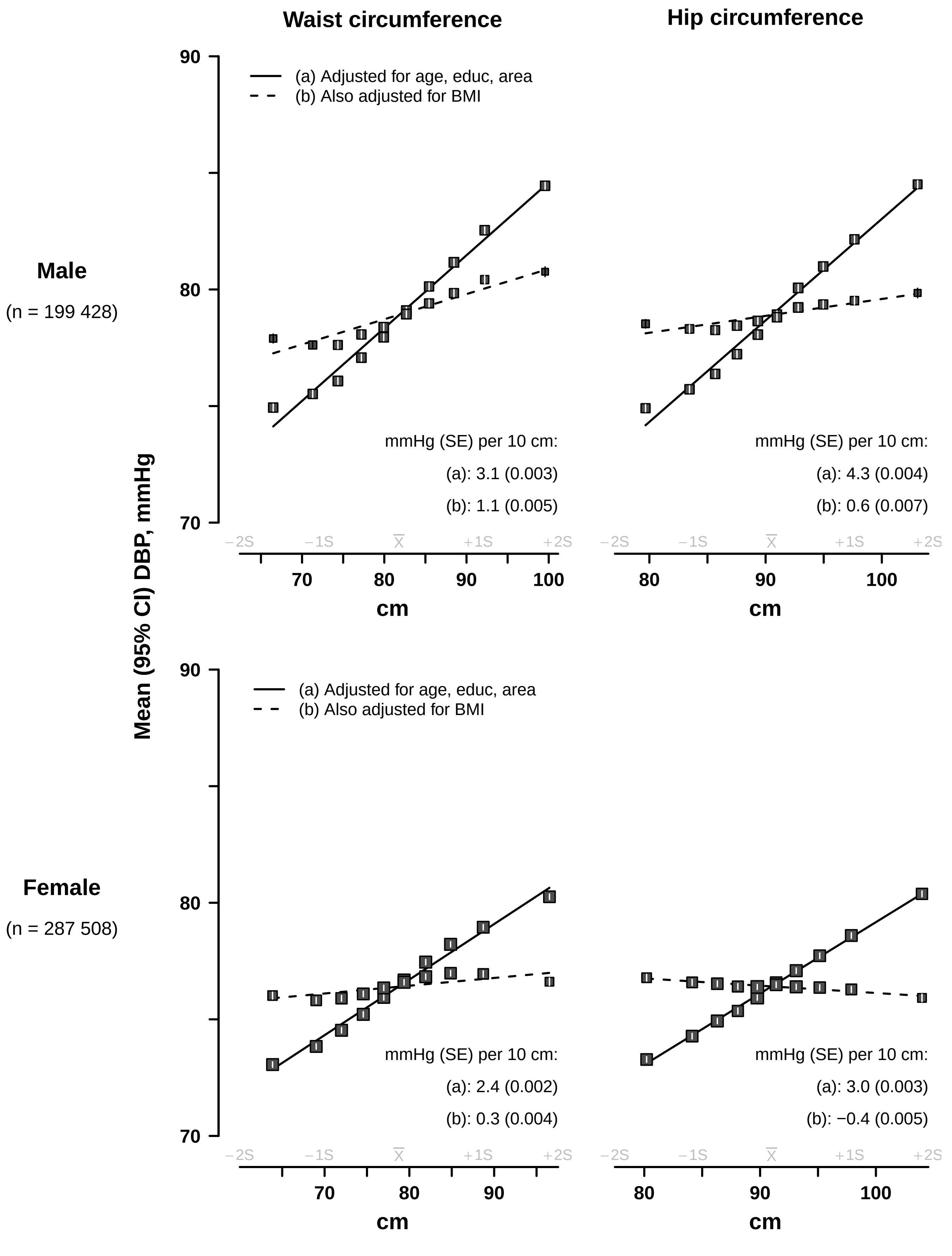

K:\kadoorie\Analysis\Adiposity Intermediate\Adip SBP\Outputs from meeting on\20140519\Web figure 3 DBP vs WC HC 19May2014.pdf

Web figure 4. Diastolic blood pressure versus waist−hip ratio and body fat percent, before (solid lines) and after (dashed lines) adjustment for BMI or waist circumference

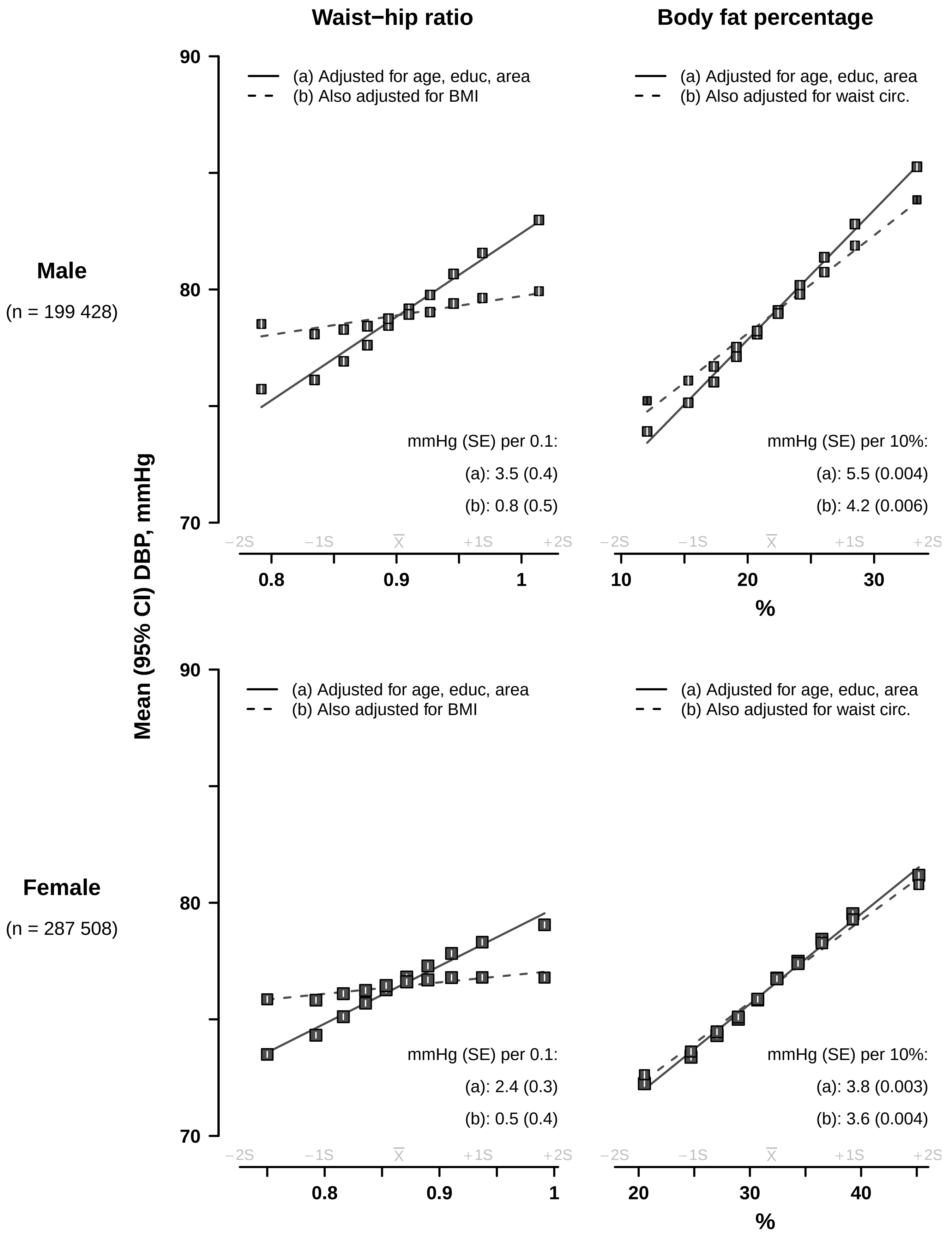

K:\kadoorie\Analysis\Adiposity Intermediate\Adip SBP\Outputs from meeting on\20140519\Web figure 4 DBP vs WHR Fatpct 19May2014.pdf

Web figure 5. Higher DBP per standard deviation of each adiposity measures among 199 428 males

Adiposity measure (SD)	mmHg per SD (95% CI)
Adjusted for age, education, area	
Height−adjusted weight (10.8 kg)	3.8 (3.78, 3.90)
BMI (3.2 kg/m²)	3.2 (3.11, 3.21)
Waist circumference (9.7 cm)	3.0 (2.96, 3.06)
Waist−height ratio (0.055)	2.8 (2.72, 2.82)
Hip circumference (6.7 cm)	2.9 (2.85, 2.95)
Waist−hip ratio (0.064)	2.2 (2.18, 2.28)
Body fat percentage (6.2%)	3.4 (3.35, 3.44)
Fat mass (6.1 kg)	3.6 (3.59, 3.69)
Fat mass index (2.1 kg/m²)	3.5 (3.43, 3.53)
Height−adjusted fat mass (6.1 kg)	3.6 (3.59, 3.70)
Additionally adjusted for waist and hip circumference	
Height−adjusted weight (10.8 kg)	3.2 (3.10, 3.37)
BMI (3.2 kg/m²)	2.2 (2.11, 2.31)
Body fat percentage (6.2%)	2.6 (2.49, 2.64)
Fat mass (6.1 kg)	3.6 (3.50, 3.69)
Fat mass index (2.1 kg/m²)	3.0 (2.93, 3.10)
Height−adjusted fat mass (6.1 kg)	3.6 (3.49, 3.69)
Additionally adjusted instead for BMI	
Waist circumference (9.7 cm)	1.1 (0.96, 1.16)
Waist−height ratio (0.055)	0.4 (0.32, 0.51)
Hip circumference (6.7 cm)	0.5 (0.39, 0.56)
Waist−hip ratio (0.064)	0.5 (0.43, 0.55)

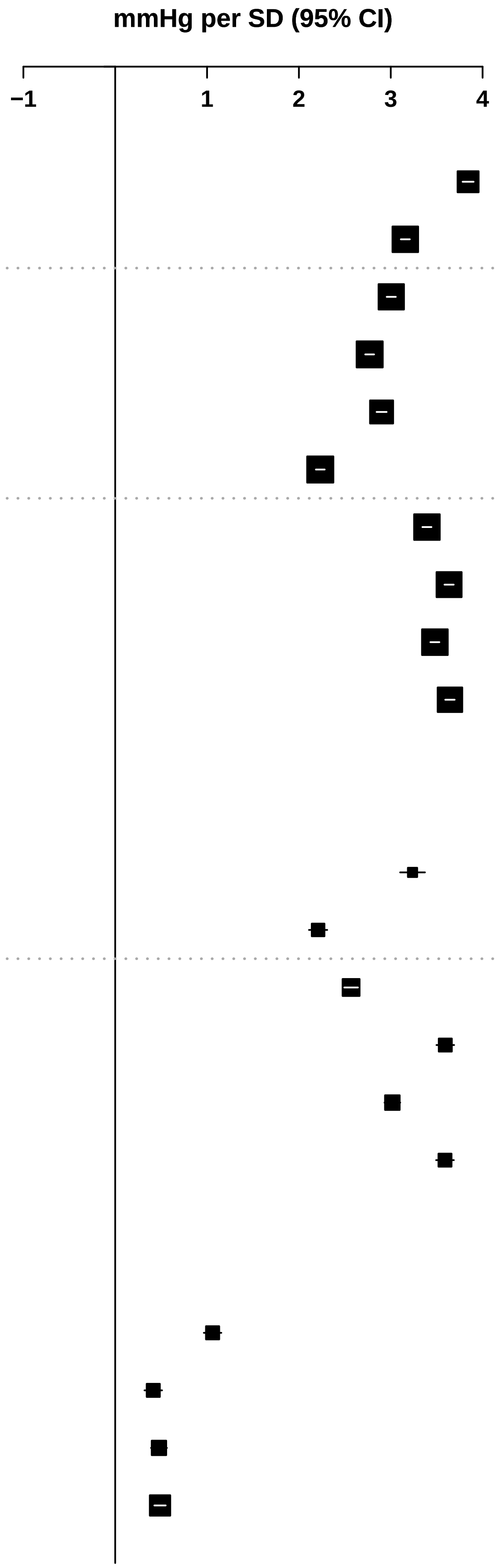

Web figure 6. Higher DBP per standard deviation of each adiposity measures among 287 508 females

Adiposity measure (SD)	mmHg per SD (95% CI)
Adjusted for age, education, area	
Height−adjusted weight (9.4 kg)	2.9 (2.84, 2.93)
BMI (3.4 kg/m²)	2.5 (2.47, 2.54)
Waist circumference (9.4 cm)	2.2 (2.17, 2.25)
Waist−height ratio (0.061)	2.2 (2.13, 2.21)
Hip circumference (6.8 cm)	2.1 (2.01, 2.10)
Waist−hip ratio (0.069)	1.7 (1.64, 1.73)
Body fat percentage (7.0%)	2.7 (2.65, 2.72)
Fat mass (6.8 kg)	2.7 (2.68, 2.76)
Fat mass index (2.8 kg/m²)	2.7 (2.63, 2.70)
Height−adjusted fat mass (6.8 kg)	2.8 (2.71, 2.79)
Additionally adjusted for waist and hip circumference	
Height−adjusted weight (9.4 kg)	3.2 (3.13, 3.33)
BMI (3.4 kg/m²)	2.4 (2.35, 2.50)
Body fat percentage (7.0%)	2.6 (2.52, 2.64)
Fat mass (6.8 kg)	3.4 (3.33, 3.49)
Fat mass index (2.8 kg/m²)	2.8 (2.76, 2.90)
Height−adjusted fat mass (6.8 kg)	3.4 (3.33, 3.49)
Additionally adjusted instead for BMI	
Waist circumference (9.4 cm)	0.3 (0.25, 0.40)
Waist−height ratio (0.061)	0.0 (−0.07, 0.08)
Hip circumference (6.8 cm)	−0.2 (−0.25, −0.11)
Waist−hip ratio (0.069)	0.3 (0.27, 0.37)

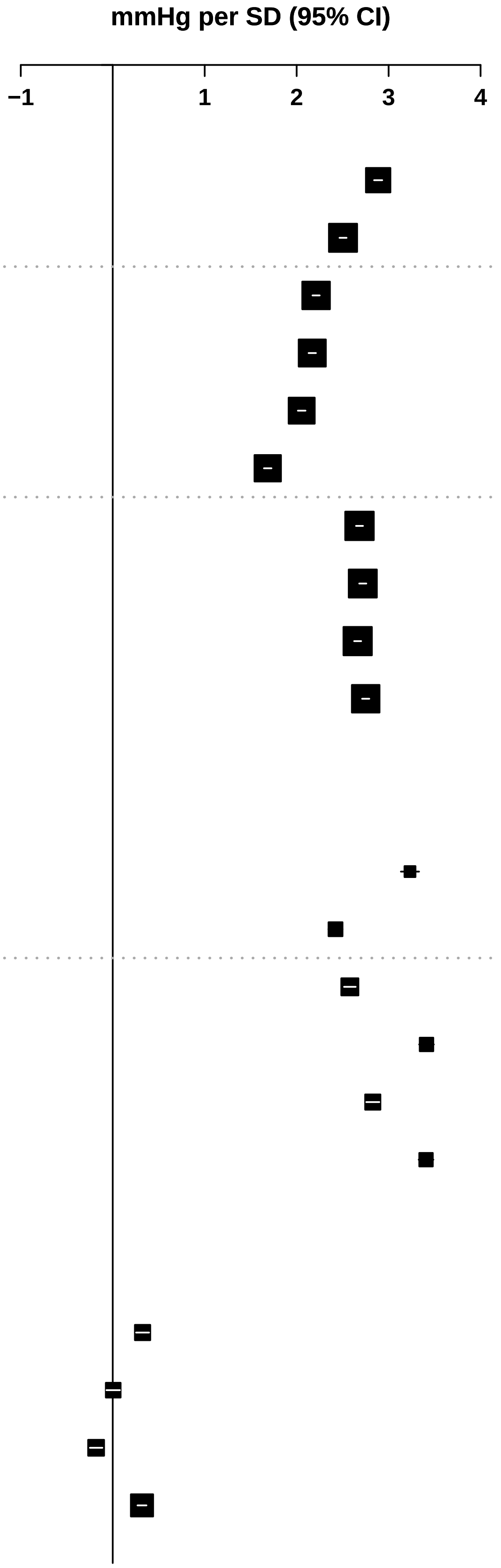

K:\kadoorie\Analysis\Adiposity Intermediate\Adip SBP\Outputs from meeting on\20130207\Web figure 6 DBP per SD female 20Feb2013.pdf

Web figure 7. Higher DBP per 10 kg/m² of BMI by different personal characteristics

Category	N	mmHg per 10 kg/m² (95% CI)
**Sex**		
Male	199 428	9.8 (9.7−10.0)
Female	287 508	7.4 (7.2− 7.5)
Heterogeneity P <.0001		
**Age**		
< 40 years	76 423	9.0 (8.7− 9.2)
40 − 49 years	149 128	9.8 (9.6−10.0)
50 − 59 years	148 909	8.4 (8.2− 8.5)
60 − 69 years	82 879	7.1 (6.8− 7.3)
>= 70 years	29 597	5.9 (5.6− 6.3)
Heterogeneity P <.0001		
**Area**		
Harbin (Urban)	54 131	9.7 (9.5−10.0)
Qingdao (Urban)	33 922	7.7 (7.4− 8.0)
Suzhou (Urban)	51 119	8.8 (8.6− 9.1)
Liuzhou (Urban)	45 302	8.1 (7.8− 8.4)
Haikou (Urban)	28 050	8.1 (7.8− 8.5)
Zhejiang (Rural)	56 357	8.1 (7.9− 8.4)
Hunan (Rural)	55 407	8.7 (8.4− 9.0)
Henan (Rural)	60 237	8.6 (8.3− 8.8)
Sichuan (Rural)	54 468	7.5 (7.2− 7.7)
Gansu (Rural)	47 943	7.5 (7.2− 7.9)
Heterogeneity P <.0001		
**Alcohol consumption**		
Non−drinker	221 113	7.7 (7.6− 7.9)
Ex−drinker	7 995	8.9 (8.1− 9.7)
Occasional	156 723	8.5 (8.4− 8.7)
Monthly	16 918	10.2 (9.7−10.7)
Reduced intake	10 544	9.1 (8.5− 9.7)
Weekly	73 643	9.4 (9.2− 9.7)
Heterogeneity P <.0001		
**Smoking**		
Never	301 094	7.7 (7.5− 7.8)
Occasional	27 769	10.2 (9.8−10.6)
Ex−regular	27 707	8.3 (7.9− 8.7)
Current regular	130 366	9.6 (9.4− 9.8)
Heterogeneity P <.0001		
**Physical activity MET quintile**		
1 (least active)	97 383	7.8 (7.6− 8.0)
2	97 387	8.1 (7.9− 8.3)
3	97 395	8.6 (8.4− 8.8)
4	97 385	8.5 (8.3− 8.7)
5 (most active)	97 386	9.2 (9.0− 9.4)
Heterogeneity P <.0001		

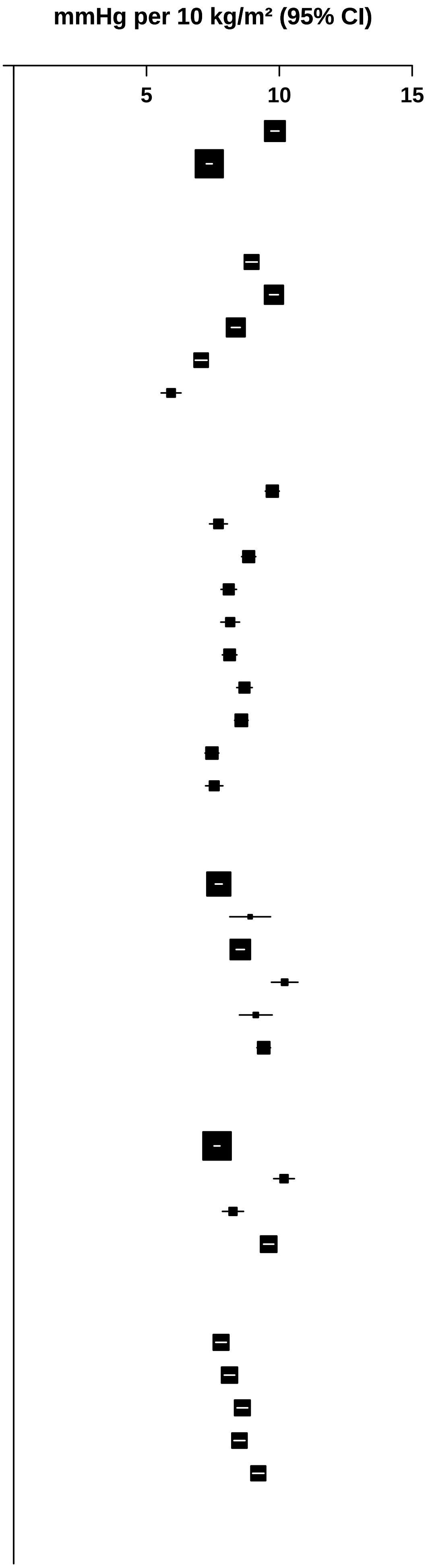

Adjusted for age, area, education, and sex as appropriate.
